# Can Financial Economics Cure Cancer?

**DOI:** 10.1007/s11293-021-09704-7

**Published:** 2021-04-01

**Authors:** Andrew W. Lo

**Affiliations:** Sloan School of Management, Massachusetts Institute of Technology, 100 Main Street, E62–618, Cambridge, 02142 MA US

**Keywords:** Drug development, Biotechnology, Venture capital, Innovation, Healthcare economics, Portfolio theory, Securitization, Investments, G10, G11, G24, G32, L26, L65, O31

## Abstract

Funding for early-stage biomedical innovation has become more difficult to secure at the same time that medical breakthroughs seem to be occurring at ever increasing rates. One explanation for this counterintuitive trend is that increasing scientific knowledge can actually lead to greater economic risk for investors in the life sciences. While the Human Genome Project, high-throughput screening, genetic biomarkers, immunotherapies, and gene therapies have made a tremendously positive impact on biomedical research and, consequently, patient lives, they have also increased the cost and complexity of the drug development process, causing many investors to shift their assets to more attractive investment opportunities. This suggests that new business models and financing strategies can be used to reduce the risk and increase the attractiveness of biomedical innovation so as to bring new and better therapies to patients faster.

## Introduction

It’s a pleasure and an honor for me to serve as President of the International Atlantic Economic Society. I want to begin by thanking Katherine Virgo and the IAES leadership for giving me this opportunity to address today’s conference attendees.

I have to begin with a disclaimer. I am no biomedical expert by any means. In fact, I’m not even a healthcare economist. My stock and trade are in applying mathematical and statistical models to investing, risk management, financial stability, and other issues in financial economics. I became interested in healthcare for purely personal reasons—friends and family members dealing with various kinds of cancer. It was through that process that I came to understand that financial economics plays a tremendously important role in healthcare, sometimes too big a role. This theme, and what we can do to change it, is what I hope to share with you today.

## Biomedicine Is At An Inflection Point

Let me start with the simple observation that biomedicine is at an inflection point. Here’s an example. In August 2015, James Earl Carter, former president of the United States, issued a press release announcing that he was suffering from stage 4 melanoma, a form of skin cancer that had metastasized to his liver and his brain. Stage 4 cancer is the most advanced form; there is no stage 5. Stage 5 is the morgue.

Upon reading this sad announcement, I assumed that fairly shortly thereafter, we would be witnessing a funeral at Arlington National Cemetery to bury our 39th president, but that’s not what happened. What did happen a few months later was nothing short of a miracle. In December, President Carter announced that he was free of cancer. All his tumors were gone. In fact, by March 2016, he announced that he was no longer seeing his oncologist. As far as his doctors were concerned, he was “cured” of one of the deadliest forms of cancer, and at the very last stage of its course.

For those of you who have ever dealt with this terrible affliction, you know that oncologists rarely ever use the word “cure” because cancer can mutate and return with a vengeance, but they’re now starting to use this word more often. While this is a remarkable story, I can assure you that President Carter is not unique in this respect. Many people today are actually being cured of cancer. This is what I’m referring to when I say that biomedicine is at an inflection point.

Three of my MIT colleagues (former president Susan Hockfield, Koch Institute director Tyler Jacks, and Nobel Prize-winning biologist Phil Sharp) published a report in 2016 that describes this inflection point. The title of their report is *Convergence: The Future of Health* (Hockfield et al., 2016). Over the past decade, a confluence of breakthroughs in the physical sciences, life sciences, and engineering has yielded a number of new therapies that have saved the lives of patients like President Carter and many others.

This convergence has often been characterized as the so-called “omics” revolution: genomics (the study of the specific sequences of our deoxyribonucleic acid (DNA)); epigenomics (the study of the on/off switches that cause certain genes to be expressed and others to be suppressed); transcriptomics (the study of how these DNA sequences are translated into proteins that make life possible); proteomics (the study of the 20,000–25,000 proteins that make up the human body); metabolomics (the study of the various chemical reactions necessary for us to maintain our health); and, most recently, microbiomics (the study of the bacterial ecosystems inhabiting our bodies). All of these “omics” have experienced dramatic advances over the last decade, with the exception of one. The “omics” that has lagged behind is econ*omics*, the study of the business models and financing strategies needed to pay for all of these amazing therapeutics.

This isn’t to say that financial economics has experienced no progress. On the contrary, many financial innovations have fueled the growth of a number of sectors. However, in the biopharma sector, the business models and funding strategies have not kept pace with the rapid pace of innovation, creating funding shortages on occasion, and typically at the early stages of preclinical drug discovery. Better ways of funding all of these amazing therapeutics is what I want to focus on today.

## Risk and the Valley of Death

Scientists and other stakeholders in this field often refer to something they call the “Valley of Death,” the stage of research and development (R&D) between fundamental scientific research and human clinical trials where funding is particularly scarce. At the later stages of drug development, there’s considerably more capital available. By the time a drug candidate has successfully gone through multiple rounds of clinical testing (e.g., to the conclusion of a phase 2 clinical trial where the results show significant efficacy in treating the disease in question), it’s said to have been “de-risked,” at which point billions of dollars pour in to complete the clinical testing process and bring the drug to market. However, to take an idea from its inception in the laboratory all the way into the clinic (i.e., to translate basic scientific research into an effective therapy) is very difficult. There isn’t nearly as much money for translational medicine as there is for late-stage drug development, hence the term “Valley of Death.”

The question I asked when I first learned about this phenomenon is “why?” Why is it the case that, in the midst of all these amazing breakthroughs, it’s so difficult to get funding for early-stage drug discovery? It turns out that the answer, at least the one I’ve come to, is economics and more specifically, the impact of increasing risk and uncertainty on drug development.

To unpack this answer, we must first briefly review how drugs are developed, a process that I’ve come to appreciate because of its underlying economic logic. The process starts with basic science in the laboratory. Once scientists understand a particular disease mechanism and wish to develop a drug to disrupt that mechanism, they need to test out their proposed method of disruption in human subjects. For obvious ethical reasons, before human trials are allowed, a drug candidate’s various properties must first be studied in Petri dishes, and then in animals like rats, dogs, or primates. If all goes well with these animal studies, then at some point scientists will feel confident enough to ask the U.S. Food and Drug Administration (FDA) for permission to administer the drug candidate to human subjects.

This is known as a phase 1 clinical trial, which involves recruiting a small number of volunteers to help assess the safety and maximum tolerable dose of the drug candidate (at this early stage, whether the candidate is effective in treating the disease is not the focus). If this phase goes well and the side effects, if any, are tolerable, a phase 2 trial is initiated in which a larger group of volunteers suffering from the disease targeted by the candidate are recruited to study the candidate’s efficacy. If the candidate is shown to be effective, then a much larger phase 3 trial is initiated to determine whether the efficacy still holds in larger and more heterogeneous populations. If safety and efficacy are established in this larger population, then the candidate becomes an approved drug. Such a process typically takes 5 to 10 years or longer, thousands to tens of thousands of volunteers, and several hundred million to billions of dollars.

Part of the reason for this lengthy and expensive process is to ensure that human subjects are never exposed to unnecessary risks. However, the result is the fact that drug development is characterized by three properties: (1) it’s very costly; (2) it takes a long time; and (3) the historical probability of success is generally very low (in the case of cancer clinical trials, the historical success rate is less than 5%). Together, these three features make it extra difficult to develop a drug. From an economic and financial perspective, they make it extra difficult for investors to fund this process from beginning to end and it’s getting worse.

**Fig. 1:**
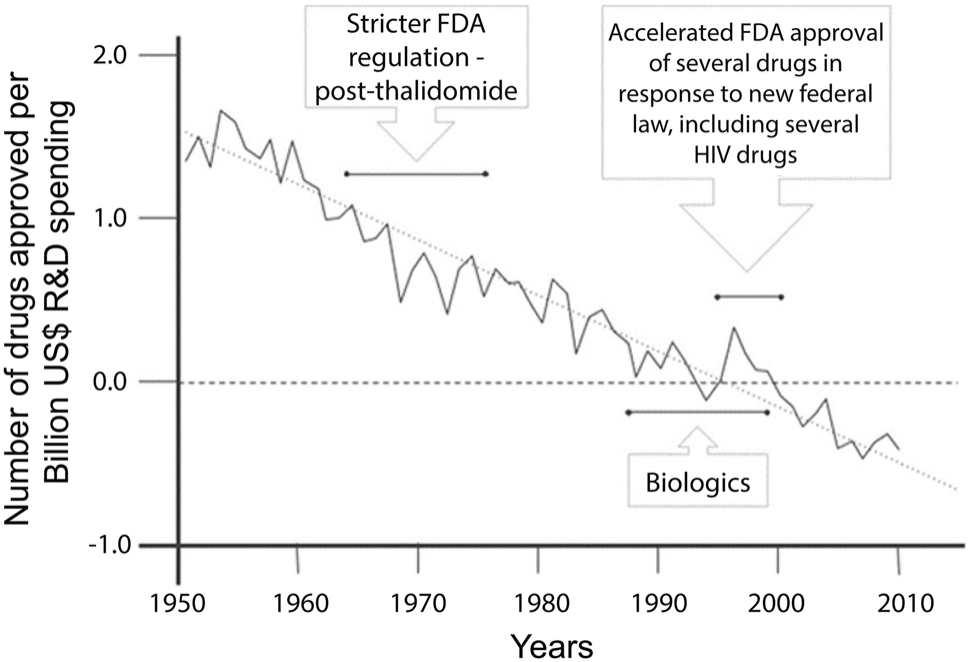
Eroom’s Law. Source: Scannell et al. (2012)

Biomedical experts have coined a term for this troubling trend: Eroom’s Law (Scannell et al., 2012). This is an empirical relationship involving the number of new drugs approved each year by the FDA divided by the total amount of R&D spending by the pharma industry (Fig. 1). This ratio captures the number of new drugs per billion dollars of R&D spending and can be viewed as a measure of the efficiency of the drug development process. The fact that this measure has been trending downward since the 1950s is cause for concern.

In case you’re wondering who Professor Eroom is, there is no Professor Eroom. “Eroom” is “Moore” spelled backwards. This graph is the opposite of Moore’s Law, the empirical relation that predicts the doubling every 18 months of the number of transistors we can fit on a chip. In contrast to the semiconductor industry, drug development seems to be getting less efficient over time. Because Fig. 1 is graphed on a semi-logarithmic scale, it really is the opposite of Moore's Law.^1^

Why is it becoming exponentially harder, year after year, to develop new drugs? Aren’t we in the midst of the “omics” revolution? Ironically, it turns out that as we get smarter in biology, drug development can become harder. This is very counterintuitive, especially to a financial economist. When studying the value of a particular company or trying to price a financial asset like a derivative security, as we collect more information and get smarter, the valuation challenge actually gets easier. This doesn’t hold true in biomedicine.

To understand the logic of this surprising result, consider the case of combination therapies, in which several drugs are used simultaneously to treat a given disease. The most well-known example of a combination therapy is the acquired immunodeficiency syndrome (AIDS) cocktail treatment, five anti-retroviral therapies that aren’t particularly effective individually, but when taken together, turn a deadly disease into a manageable chronic condition. Once scientists discovered that combinations can work where individual therapies don’t, it led to a number of combination therapies, including the therapy that Jimmy Carter received.^2^ Now that we’ve become smarter and realize that combination therapies can work where individual drugs don’t, drug development should get easier, shouldn’t it? Let me explain why this isn’t necessarily the case.

There are currently approximately 2,800 approved drugs on the market today. How hard would it be to search through these drugs to find a pair that might be effective in treating a given disease? In fact, a number of biomedical experts believe that we already have all the drugs we need to treat any disease and just need to find the right combinations. However, with 2,800 drugs to choose from, there are 3,918,600 unique pairs. Each requires a separate and independent series of clinical trials to evaluate safety and efficacy, which will take many years and many millions of dollars, and with generally very low probabilities of success. If we consider triplets, quadruplets, or, as in the case of the AIDS cocktail, quintuplets, as well as other variables such as dosage regimens and biomarkers, it’s clearly impossible to do any kind of systematic search across all combinations, despite the fact that, from a scientific and ethical perspective, it makes sense to examine these possibilities.

The fact that we’ve become smarter has greatly increased the complexity of drug discovery which, in turn, has implications for financial risk. For example, the number of ways that young upstart biotech companies can now render existing franchises of big pharma completely obsolete by developing more effective combination therapies has greatly increased. If there’s one thing financial economics has taught us, it’s that investors don’t like risk and they vote with their dollars.

## The Sharpe Ratio

**Fig. 2:**
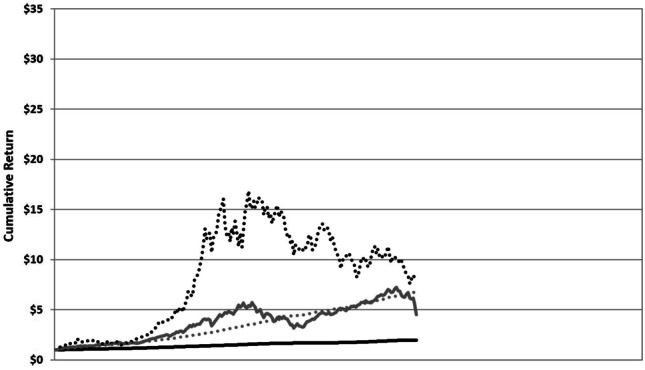
Four financial investments with different risk/reward characteristics over an unspecified multi-year investment horizon

Just so we understand what we’re up against when dealing with investor risk preferences, let me offer an illustration that I use with first-year master of business administration (MBA) students in my introductory finance class. I ask them to choose one of four different financial investments displayed in Fig. 2. I don’t tell them what they are or even over what time period they span. I simply show them what happens if you invest a dollar in each of these assets at the outset and leave it over this multi-year investment horizon.

Each of these investments has a different risk–reward tradeoff. The solid black line turns a dollar into two dollars over this unspecified investment period; not very interesting, but not particularly risky. The solid gray line turns a dollar into five dollars, more rewarding, but more volatile. The dotted black line is the most rewarding of all, turning a dollar into about $7.50, but with far more volatility. Finally, the dotted gray line is somewhere in the middle, turning a dollar into about $6.50, not nearly as rewarding as the dotted black line, but not nearly as risky. Please take a minute now to make a choice before reading on.

It turns out that, among the vast majority of audiences to which I’ve presented this series of investments, the most popular choice by far is the dotted gray line because it seems to have the best tradeoff between risk and reward. I suspect this is what you chose as well.

**Fig. 3:**
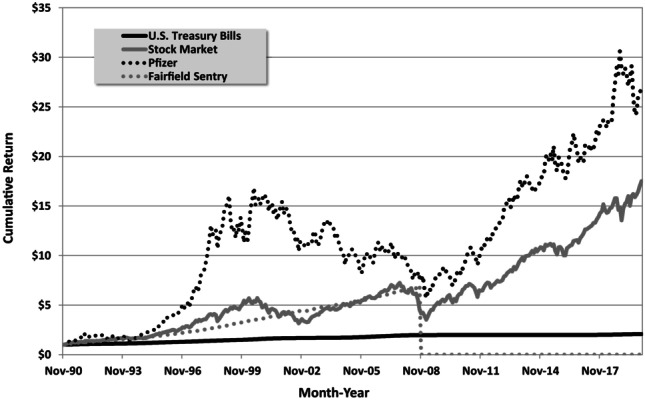
Performance of four financial investments revealed

Let me now reveal what the four investments are (Fig. 3). First of all, the time period is from 1990 to 2008. The solid black line is U.S. Treasury bills, the safest asset in the world (at least until the next budget impasse), but not very rewarding. Had you put your money into T-bills in 2008, you would have earned pretty much nothing since then.

The solid gray line is the Standard and Poor’s (S&P) 500; most of us already invest in this asset through our retirement funds, yet each year I run this poll, only one or two of my MBA students choose it. Had you invested in the U.S. stock market in 2008, congratulations, you would have done just fine since then.

The dotted black line is the pharmaceutical company Pfizer; much more volatile but had you invested in 2008, you would have outperformed the S&P 500 by a significant margin. Only a handful of students (budding entrepreneurs and hedge-fund managers, no doubt) choose this option.

What about the most popular choice, the dotted gray line? This is the performance of the Fairfield Sentry Fund, a private investment vehicle that served as a feeder fund for the Bernie Madoff Ponzi scheme. Investors in this fund lost everything in 2008 when the scheme collapsed, which is why I had to end my example in 2008.

This is a classic illustration of human nature. We’re all drawn to high-yielding, low-risk assets. In financial economics, we have a concept for describing this tendency: the Sharpe ratio, which is defined as the excess expected return above the risk-free asset divided by the risk (as measured by the standard deviation). During the sample period, the Sharpe ratios of Pfizer and the S&P 500 were 0.43 and 0.54, respectively, whereas the Sharpe ratio of the Madoff Ponzi scheme (on paper, before it blew up) was 2.98, an order of magnitude larger than the other two. This is how Madoff was able to extract $50 billion of wealth from unsuspecting investors, who were mesmerized by Madoff’s high Sharpe ratio.

As the “omics” revolution has increased the complexity and risk of biomedical assets, the Sharpe ratios of those assets have declined, other things equal, implying that capital will shift to lower-hanging fruit (e.g., technology startups). This trend will be especially pronounced in the riskiest parts of the industry (i.e., the Valley of Death).

## Risk and Reward of Biomedical Assets

Now consider another investment opportunity, this one involving a single asset that requires a $200 million upfront investment, will take 10 years before any return is generated, and has a probability of a positive cashflow of 5% (in other words, a 95% failure rate in which the payoff is $0). Would you be willing to invest in this opportunity?

When I present this investment to my students, I usually get no takers. Every so often, a particularly intrepid student will raise their hand and ask, “in the 5% outcomes, can you tell us what the payoffs are?” Most investors don’t even bother asking. When they see numbers like $200 million upfront, a 10-year holding period, and a 95% failure rate, they have no interest.

**Fig. 4:**
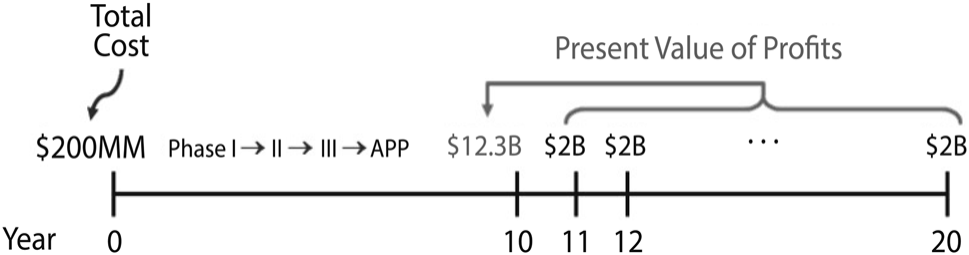
Stylized risk–reward profile of a typical anti-cancer drug development program

These are the back-of-the-envelope numbers for what it takes to develop and test a typical anti-cancer compound: a present value of $200 million in out-of-pocket costs, 10 years of clinical trials from phase 1 to 3, then FDA review, and an overall success rate of 5%.^3^ In the unlikely event that this program does succeed, an anti-cancer drug will generate profits of about $2 billion a year in years 11 to 20, which amounts to a present value of $12.3 billion in year 10 using a 10% cost of capital, which is typical for the pharmaceutical industry (Fig. 4).

Let me restate this investment opportunity: an upfront payment of $200 million, a 10-year investment horizon, and then a 5% probability of a $12.3 billion payoff in year 10 or a 95% probability of nothing. Would you invest? These parameters imply an expected annualized rate of return of 11.9% during the investment horizon and an annualized standard deviation of 423.5%, a Sharpe ratio of 0.02. This is why so few investors are interested. It’s simply too risky a proposition.

## Finance to the Rescue

This is the nature of the challenge in the biopharma industry, and it’s also where financial economics may be able to play a useful role. I learned this in a very personal way years ago, when my mother was battling non-small cell lung cancer. She had exhausted the standard of care, nothing seemed to be working, so we started looking into various clinical trials. An MIT colleague introduced me to a very successful biotech company developing a number of anti-cancer therapies, including one for non-small cell lung cancer. I was privileged to meet with the chief scientific officer, who brought along his chief financial officer (CFO). I asked the two of them what I thought was a relatively straightforward question: “Does your source of financing have any influence on your scientific agenda?” I was quite interested in that agenda, because one of the projects on their list might have been able to help my mother. The chief scientific officer shook his head ironically, looked at his CFO, looked back at me, and said, “Influence?? Our funding drives our scientific agenda.”

I was absolutely shocked by that response. As an economist, I get it (you have to pay for stuff), but as the son of a dying patient, I was absolutely outraged. What does stock market volatility, Fed policy, or interest rates have to do with whether you should treat cancer by angiogenesis inhibitors or immunotherapy? Nothing. Yet these financial considerations often drive even a successful company’s scientific agenda.

As I said at the outset, I’m not a biomedical expert, but shouldn’t the science be driving the financing instead of the other way around? I think we have it backwards. That’s what started me down this path of trying to understand whether we can do better for patients by using different types of financing. I think we can. Financial engineering can play a significant role in turning this around, allowing the science to drive the financing.

Let me give you an example. Suppose that instead of focusing on one anti-cancer program, we invest in 150 of them, all at the same time. Now, I know it’s going to take 150 × $200 million or $30 billion of capital to do this. Where are we going to get $30 billion? Economists have a very simple answer to this question: assume we have $30 billion.

I realize this may be less than satisfying to non-economists, but I’ll come back to this shortly. If we did manage to raise $30 billion, then consider the investment profile of a fund investing in 150 mutually independent projects, each with the risk–reward profile of the project described earlier (we’ll return to the independence assumption later as well). It turns out that the economics of this business proposition are entirely different.

As a consequence of diversification, the expected return is the same 11.9%, but the standard deviation becomes 34.6%, yielding a Sharpe ratio of 0.34. Would you be willing to invest in this opportunity? Many more investors would and that’s where the $30 billion will come from; from investors who are attracted to the much-improved performance prospects of a highly diversified portfolio of mutually uncorrelated anti-cancer programs.

Now, a number of questions are prompted by this overly simplistic analysis of the biopharma industry, but perhaps the most immediate is, “Can we really raise $30 billion from investors?” The answer is, “It depends.” It depends on the underlying risk–reward profile of these 150 assets. If the assumption of independently and identically distributed (IID) projects with the risk–reward profile of Fig. 4 is correct, not only can we raise the $30 billion, we can raise it from debt markets, by issuing anti-cancer bonds. Think about that. Typically, early-stage drug development is financed through venture capital (VC) or public equity via an initial public offering (IPO), not by fixed-income instruments, but debt markets have two to three orders of magnitude more capital than VC and public equity markets combined.^4^

How much debt can we issue? Again, it depends on the portfolio. With 150 IID projects, each with a 5% probability of success, the probability of three successes out of 150 “shots on goal” (to use a hockey or soccer term) is 98.18% according to the binomial distribution. Therefore, such a portfolio should be worth at least 3 × $12.3 billion = $36.9 billion with 98.18% probability in year 10. If we finance the cost of this portfolio by issuing 10-year pure discount bonds with a face value of $36.9 billion, the probability of default is simply 1–0.9818 = 0.0182, or 1.82%. This corresponds to a historical default rate of single-A-rated debt.^5^ As of 31 January 2021, the average yield on single-A debt was 1.64% (Federal Reserve Bank of St. Louis, 2021). At this yield, our bond issue would raise $31.3 billion, more than sufficient to fund the 150 programs (Table 1).

**Table 1:** Amount of debt financing available for funding a portfolio of 150 independently and identically distributed anti-cancer drug programs

Event	Probability	Minimum Year-10 NPV	Maximum Year-0 Proceeds at 1.56% (BofAML AA 10-Yr as of 1/31/21)	Maximum Year-0 Proceeds at 1.64% (BofAML A 10-Yr as of 1/31/21)	Maximum Year-0 Proceeds at 2.16% (BofAML BBB 10-Yr as of 1/31/21)
At least 1 hit:	99.95%	$12,289	$10,527	$10,444	$8,501
At least 2 hits:	99.59%	$24,578	$21,054	$20,888	$17,003
At least 3 hits:	98.18%	$36,867	$31,580	$31,333	$25,504
At least 4 hits	94.52%	$49,157	$42,107	$41,777	$34,005
At least 5 hits:	87.44%	$61,446	$52,634	$52,221	$42,507

Bond yields obtained from the Federal Reserve Bank of St. Louis (2021) FRED website

Using other financial engineering tools such as securitization,^6^ credit default swaps, and other derivative securities, we may be able to do even better, especially given that corporate bond yields are currently near historic lows. There’s a large amount of investor capital seeking attractive investment opportunities.

At this point, many readers may be questioning the wisdom of this approach. After all, securitization and the associated alphabet-soup of financial securities figured prominently in the 2008 financial crisis. Why would we want to employ the same techniques here?

In fact, it was studying the 2008 crisis that motivated me to follow this line of thinking. The financial crisis occurred not because these techniques didn’t work, but because these techniques worked far too well. Securitization is an extraordinarily powerful tool for raising large amounts of capital over a short period of time to focus on very specific purposes. Imagine if some of those purposes included curing cancer, Alzheimer’s, and the many other diseases that afflict patients today. Imagine if those purposes included some of the biggest challenges facing humankind, such as climate change, the looming energy crisis, space colonization, or decaying infrastructure.

**Fig. 5:**
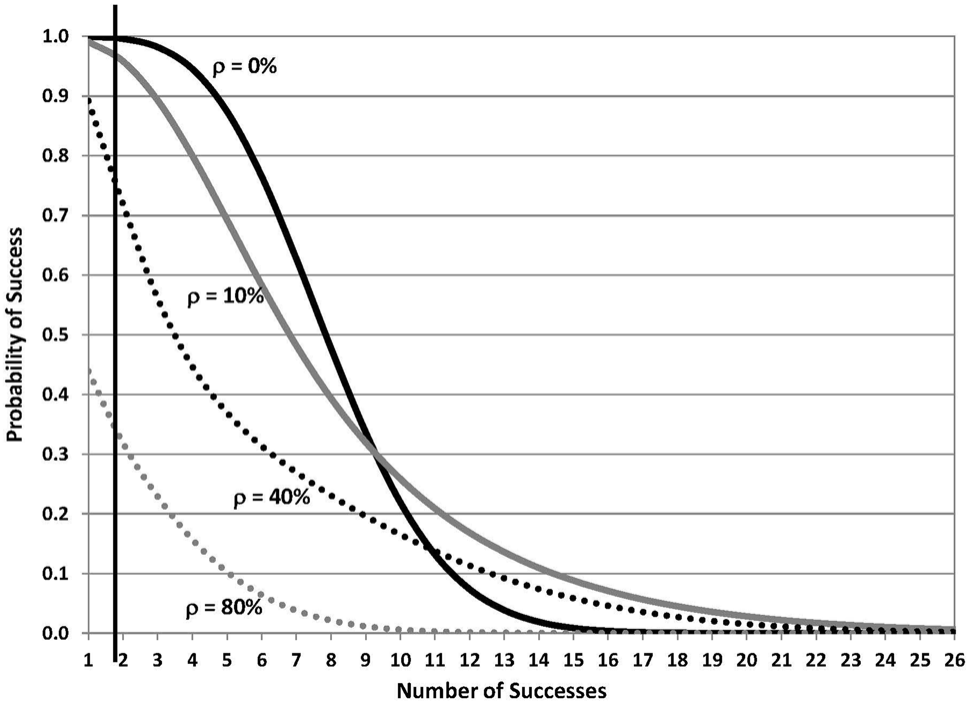
Probability of at least *k* successes out of 150 Bernoulli trials, each with a 5% probability of success, for various assumptions of pairwise correlation of success: 0% (solid black), 10% (solid gray), 40% (dotted black), and 80% (dotted gray)

Of course, we do have reason to be wary. For example, one of the key lessons of 2008 is the critical role that correlation played in creating unanticipated losses in the subprime mortgage market. The same applies in the biomedical context. If our assumption of independence is correct, then the probability of at least three successes out of 150 programs is indeed 98.18% as the black curve in Fig. 5 confirms. However, if we assume that the failures among the 150 programs are pairwise correlated at 10%, the gray curve shows that the probability of at least three successes declines to 89.3%. This value declines further to 56.1% with 40% correlation (dotted black curve), and to 22.9% with 80% correlation (dotted gray curve). At these levels of default correlation, debt financing is impossible (as subprime mortgage lenders discovered starting in 2006).

Therefore, the key to unlocking the tremendous pools of capital in corporate debt markets is to manage the correlation of the portfolio, something financial economists have understood since Markowitz’s (1952) path-breaking thesis on mean–variance portfolio optimization. The analogy used by an associate of mine who is a seasoned pharma industry executive is the comparison between his daughter’s third-grade soccer team, where the players all crowd around the ball, and World Cup soccer, where the players are properly spread out.

From his perspective, the pharma industry is more like third-grade soccer. They tend to crowd around the same ideas rather than diversifying. Of course, there are compelling scientific reasons for pursuing similar therapeutic modalities. The current focus in oncology on immunotherapies is a case in point. Scientific breakthroughs in recruiting the human body’s own immune system to fight tumor cells has led to a number of first-in-class therapies, including pembrolizumab, or Keytruda, the drug that cured President Carter’s stage 4 melanoma.

From a biomedical perspective, pursuing the most exciting therapeutic modalities that have been scientifically validated by recent research is sensible, especially given the high fixed costs associated with developing expertise in a given modality. However, from a financial perspective, the clustering of investments in the same therapeutic class does little to reduce risk. If one anti-PD1 therapy fails its clinical trial, that’s generally not positive news for similar anti-PD1 therapies in the same biopharma company’s pipeline. As a result, the Sharpe ratio of a highly correlated portfolio will attract less capital than that of a well-diversified portfolio.

## A Financial Puzzle

In fact, diversification is one of the most direct routes to a higher Sharpe ratio and lower cost of capital, and this aspect underscores a critical and puzzling feature of biomedical portfolios. Recall from the Sharpe-Lintner capital asset pricing model (CAPM) that the cost of capital of a given investment, $i$, is directly proportional to the amount of systematic risk of that investment’s return, i.e., its market beta, ${\beta }_{i}$:

1 $${\text{E}}\left[{R}_{i}\right]={R}_{f}+{\beta }_{i}\left({\text{E}}[{R}_{m}]-{R}_{f}\right) ,$$

where ${\beta }_{i}\equiv \mathrm{Cov}\left[{R}_{i},{R}_{m}\right]/\mathrm{Var}\left[{R}_{m}\right]$ captures the correlation between the asset’s return and the return on the aggregate market. Therefore, the estimated cost of capital for a given drug development program, a key input in all drug companies’ go/no-go decisions, is determined entirely by the program’s beta.

What do you think a typical drug company’s beta is? More specifically, how do the betas of early-stage drug discovery companies in the biotechnology sector compare to those of mature pharmaceutical companies?

Common intuition would suggest that the betas of early-stage biotech companies should be close to zero. After all, what do stock market fluctuations have to do with whether a cancer drug program will succeed or fail? In the case of large pharmaceutical companies, this same logic suggests that their betas should be higher, due to the fact that they have more complex business relationships, products, and services, and are, therefore, more exposed to changes in general economic conditions.

**Fig. 6:**
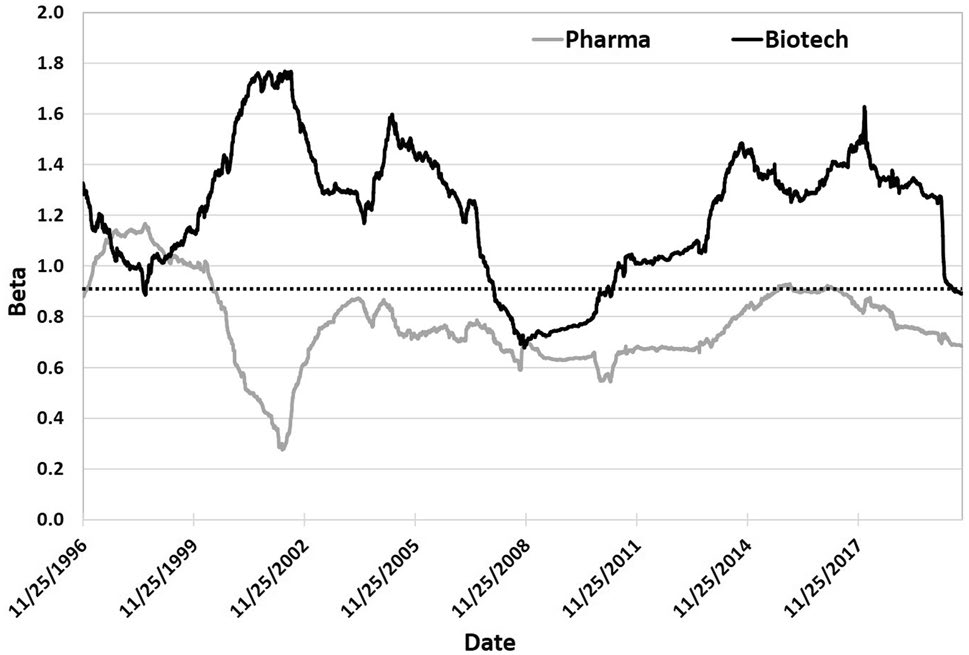
500-day rolling-window estimates of beta coefficients with respect to the S&P 500 of the NYSE Arca biotech (black line) and pharma (gray line) indexes, from 25 November 1996 to 20 September 2020

This intuition fails dramatically, as shown in Fig. 6, which contains 500-day rolling-window estimates of the betas of the New York Stock Exchange (NYSE) Arca biotech and pharma indexes from 25 November 1996 to 20 September 2020 using daily data. The biotech index betas are almost always higher than those of the pharma index, and almost always above 1.0. In contrast, the pharma betas are almost always below 1.0, confirming the Wall Street consensus that the pharma industry is less cyclical than the market, presumably because the price elasticity of demand for drugs and medical devices is less sensitive to business cycle fluctuations than other consumer goods. Table 2, which contains the betas of individual pharma and biotech companies, provides a concrete illustration of this pattern, showing that pharma companies have much lower betas and, therefore, lower costs of capital than even very successful biotech companies.^7^ Why are biotech betas so high?

**Table 2:** Estimated betas of a selection of pharmaceutical and biotech companies obtained from finance.yahoo.com on August 14, 2020

Big Pharma			Biotech		
Company	Beta	CAPM Cost of Capital	Company	Beta	CAPM Cost of Capital
Eli Lilly	0.22	5.32%	Agios	2.03	16.18%
Gilead	0.66	7.96%	Alexion	1.39	12.34%
GSK	0.40	6.40%	Alnylam	1.80	14.80%
J&J	0.69	8.14%	BridgeBio*	1.14	10.84%
Merck	0.49	6.94%	Editas	1.98	15.88%
Novartis	0.41	6.46%	Moderna**	0.10	4.60%
Pfizer	0.73	8.38%	Sarepta	1.83	14.98%
Sanofi	0.47	6.82%	Solid	1.16	10.96%

Betas for starred companies were estimated with respect to the S&P 500 index return using daily data starting from company inception date. CAPM costs of capital computed using estimated betas, assuming a risk-free rate of 4% and an expected return of the market portfolio of 10%. *estimated, 6/28/19 to 8/14/20; **estimated, 12/10/18 to 8/14/20

## Puzzle Resolved

The answer to the puzzle of big biotech betas has to do with the fact that biotech companies actually face two types of risk: scientific and financial. We’ve already discussed scientific risk, and almost by definition, such risk is indeed uncorrelated with aggregate economic fluctuations. However, if a biotech company runs out of cash, its progress comes to a full stop. Moreover, because the nature of biomedical innovation is so heavily dependent on skilled labor, the departure of key personnel due to financial disruptions can destroy significant shareholder value, often permanently. Therefore, a biotech company’s financial risk can dwarf its scientific risk (no financing, no drug); hence the outsized betas of companies in this sector.

Pharma companies are less sensitive to financial disruptions simply because they’re generating cash and usually profitable, hence they can weather the storms of recessions without interrupting their development programs. Also, given their size and franchise value, the departure of key personnel is unlikely to have the same impact as in the case of smaller biotech companies.

Another way of thinking about big biotech betas is in terms of rollover risk. Imagine a financial services company that requires cash to run its business, and finances that cash using short-term debt contracts that constantly have to be renewed. As long as the economy is doing well, rolling over this debt should not be a problem. However, as soon as an economic downturn or credit crunch occurs, the company may not be able to refinance, at which point it’ll be forced to seek bankruptcy protection from its creditors. During such a process, it’s a safe bet that most of the customers of this company will take their business elsewhere, hastening the company’s demise.

Even though a typical biotech company uses no debt financing, the principle is the same. If a company can’t finance its current operations (e.g., paying its employees, paying for ongoing clinical trials, purchasing needed equipment), it will enter a state of financial distress, at which point key employees may depart, likewise hastening this company’s demise.

An interesting piece of anecdotal evidence that supports the hypothesis of financing risk can be found in the biotech betas shown in Table 2. Notice that among all the biotech companies in that list, there is only one with a beta less than 1.0: Moderna, the company built on a messenger ribonucleic acid (mRNA) platform technology that recently received FDA approval for its coronavirus disease 2019 (COVID-19) vaccine. Its beta is only 0.10, which is statistically indistinguishable from 0. Why is Moderna’s beta so different from those of other biotech companies?

The answer lies in financing risk. Between Moderna’s launch in 2010 and just prior to its IPO in November 2018, the company raised $2.6 billion in equity financing. As of 30 September 2018, the company had cash, cash equivalents, and investments of $1.2 billion. Its IPO on 6 December 2018 was the largest in the history of the biotech industry at the time, raising another $620 million and valuing the company at $7.5 billion. All of this occurred well before the COVID-19 pandemic and Moderna’s program to develop a vaccine for the severe acute respiratory syndrome coronavirus 2 (SARS-CoV-2) target.

By raising so much cash, Moderna eliminated much of its financing risk for the next several years, giving it plenty of time and resources to develop several of its programs to completion, irrespective of general business conditions. Once the financing risk was addressed, the only remaining risk was scientific. This was already addressed at the time of the IPO by Moderna’s diversified portfolio of 21 mRNA-based drugs for a variety of diseases, of which 10 were in clinical trials. With both risks reduced, Moderna’s cost of capital should be much lower than less well-financed biotech peers, implying a much higher valuation based on standard discounted cashflow models and making it much more attractive to investors. On 18 December 2020, this financing strategy paid off handsomely: Moderna achieved its very first FDA approval for mRNA-1273.

## Can This Really Work?

I realize that my highly stylized examples raise more questions than they answer. For example, do we really need $30 billion? What exactly is the market failure, if any, in the biopharma industry? Can you really find 150 uncorrelated biomedical assets? Is it organizationally feasible to manage 150 programs at the same time? Aren’t big pharma companies already doing this? Shouldn’t the government be doing this? My short answer to these questions is, “I have no clue. I am just a financial economist!”

However, I have a much longer answer, one that I’ve been working on for the past decade. That answer is a series of research articles I’ve written in collaboration with a number of experts in the life sciences and the biopharma industry. In these articles, we simulate several new business models for funding therapeutic development programs in various types of cancer (Chaudhuri et al., 2019; Das et al., 2018; Fagnan et al., 2013; Fernandez et al., 2012), Alzheimer’s disease (Lo et al., 2014), rare diseases (Fagnan et al., 2014, 2015), and opioid addiction and pain management, for example.^8^ The answer is that, in many circumstances, better financing structures and business models are indeed possible, and when used properly, they result in more drugs and devices reaching a larger number of patients sooner.

To perform these simulations, we start with what I call the “Fundamental Law of Healthcare Finance,” a rather grandiose term for a simple expression for the economic value of a single drug development program:

2 $$\text{E[NPV] = PV[Profits]}\times\text{PoS}-\text{Costs.}$$

This equation relates the expected net present value of a drug program to just three terms: the present value of profits if the drug is approved, the program’s probability of success (PoS), and the associated development costs. This is, of course, a gross oversimplification of a much more complicated reality in which drug sales must be forecasted. Those forecasts depend on many factors, including competition, regulatory processes, drug pricing strategies, and health insurance reimbursement policies. However, Eq. (2) does capture the major features of the key drivers of drug development and why finance can play a useful role.

Of the three terms in Eq. (2), economists are quite capable of estimating costs and present values, but we have absolutely nothing to say about the probabilities of success as this is the domain of scientific and medical experts. However, the use of data to estimate PoS can provide a useful starting point for any financial analysis of drug development programs. A few years ago, we launched a project at MIT called Project ALPHA (Analytics for Life-sciences Professionals and Healthcare Advocates), which is a collaboration with one of the largest data providers to the life sciences industry, Informa, to estimate PoS using hundreds of thousands of clinical trial events going back two decades, with estimates stratified by disease group, clinical phase, with or without biomarkers, and other features.^9^ We update these estimates on a quarterly basis and make them publicly available at no charge on the Project ALPHA website (MIT Laboratory for Financial Engineering, 2021). We also provide estimates on a rolling-window basis to capture time variation in PoS, and have found that these estimates have been climbing in most disease areas over the past decade, confirming the impact of the “omics” revolution.

The complexity created by this revolution means that no one person will know all that’s needed to take an idea for therapeutic intervention from conception to an approved drug. Therefore, financial and biomedical experts have to collaborate to develop the appropriate financing structures and business models for each unique context. To facilitate that collaboration, also posted on the Project ALPHA website are our various publications in healthcare finance and the corresponding software components for running the business simulations in those publications. All the software is distributed with open-source licenses, allowing anyone to download, run, and modify the code to suit their own assumptions and use cases.

Most of these publications don’t appear in finance or economics journals, for the simple reason that there’s virtually nothing novel about the finance or economics involved, so I haven’t bothered submitting them to those journals. Ideas such as portfolio theory, securitization, and risk management have been well-known to financial economists for decades. The only novel aspect is the application of these techniques and models to the life sciences, which is why collaboration between finance and biomedicine is so important.

## Proof of Concept

A personal illustration of this process of collaboration involves my research on rare or orphan disease drug portfolios. According to the Orphan Drug Act of 1983, an orphan disease is one that affects fewer than 200,000 patients in the United States. These are diseases like hemophilia, cystic fibrosis, amyotrophic lateral sclerosis (ALS or Lou Gehrig’s Disease), Gaucher disease, Rett syndrome, and Duchenne muscular dystrophy. Even though any given orphan disease may affect only a small number of patients, there are over 7,000 different such diseases that collectively affect about 30 million Americans, more than the total number of cancer patients in the U.S.

For a variety of reasons, including small patient populations, government incentives, and a better scientific understanding of a number of these conditions, as a class these diseases have a much higher PoS than many other categories. Therefore, we don’t need $30 billion to make progress here, nor do we need 150 programs to achieve sufficient diversification. According to our simulations (Fagnan et al., 2015), $400 or $500 million and 10 or 20 programs can yield very attractive returns for investors. The reason has to do not only with the higher PoS for rare diseases, but also the fact that, because of the heterogeneity of these diseases, the success or failure of one rare disease program is almost completely unrelated to that of another. This means that the assumption of pairwise independence is actually a reasonable approximation in this use case. Given that lack of correlation, we can construct portfolios that are smaller and have more attractive risk-adjusted returns. In a collaboration with researchers at the National Institutes of Health’s rare disease division (the National Center for Advancing Translational Sciences), we published a simulation of a rare disease portfolio that generated a return of 22% with moderate risk (Fagnan et al., 2015), a surprising result that exceeds the performance of most hedge funds during the same period.

Prior to publication, I presented these results at a healthcare finance conference I co-organized at MIT called CanceRx, and it caught the attention of a former MIT student of mine, Neil Kumar, a chemical engineering Ph.D. who enrolled in my introductory finance course many years ago. He approached me after the conference and asked whether I could re-run the analysis with his own assumptions, and over the next few months, we formulated a more realistic simulation that incorporated sharper estimates of PoS, development costs, and revenue forecasts.

Those results formed the basis of a rare disease portfolio company that he and I co-founded called BridgeBio Pharma. Within the space of five years, this company raised over $1 billion in capital, and currently has a market value in excess of $7 billion, so all the investors have earned a very attractive rate of return. However, the company is most proud of the fact that they now have 21 assets in their pipeline, most of which were acquired preclinically, from the Valley of Death. Four of them are currently in phase 3 trials, with a potential approval as early as 2021. With the right kind of financing and sufficient capital, tremendous progress is possible.

## Conclusion

These simulations, case studies, and proofs of concept suggest that finance can make a tremendous difference in patients’ lives. This kind of impact is something that most of us in finance aren’t familiar with, but it’s become a personal mission and passion of mine, especially after I heard the story of Harvey Lodish, one of my MIT colleagues. After learning about his involvement with rare diseases, I decided then and there that I wanted to be Harvey Lodish. Let me explain.

Harvey’s a professor of biology at the MIT Whitehead Institute for Biomedical Research and in 1983, he was approached by a biotech VC seeking his help to develop a therapy for Gaucher disease, a rare condition caused by a single-gene mutation that prevents the body from producing an important housekeeping enzyme. Without this enzyme, fatty acids build up in several organs and the disease becomes fatal by the time the patient becomes a teenager.

Harvey and several of his colleagues agreed to join forces and in 1991, their little company succeeded in developing the very first enzyme replacement therapy known as Ceredase. Through this therapy, and its many improvements since then, the lives of hundreds of thousands of Gaucher patients have been saved. You may have heard of this company. Its name is Genzyme and in 2011, it was acquired by Sanofi for approximately $20 billion. However, that’s not why I want to be Harvey Lodish (although that’s not a bad reason!). I want to be Harvey because of what happened in 2002. In that year, Harvey’s daughter was pregnant with her second child, a boy she named Andrew, who was diagnosed in utero with Gaucher disease.

What are the chances of this happening? When Andrew turned 10 in 2012, he did develop the full-blown version of the disease, but he’s doing just fine, living a perfectly normal life thanks to the drug that his grandpa helped to develop over a decade before he was born.

When I talked with Harvey about it, it was quite an emotional conversation. I asked him whether he had any idea that Gaucher disease ran in his family. He said he had no idea whatsoever, and observed that 1983 was a full two decades before the human genome was sequenced, so it would have been quite a bit more difficult to test for this condition back then than it is today. He said never in a million years could he have dreamed that what he was working on at Genzyme would someday save his grandson’s life. He did it because of scientific curiosity and the possibility that his expertise could help some patients in desperate need.

This is why I want to be Harvey Lodish. I don’t have a Ph.D. in molecular biology. I can’t help develop the drugs that will one day save the life of my as-yet-unborn grandchildren. However, I realized a few years ago that all of us can be Harvey Lodish, if we *invest* in the drugs that will save the lives of our as-yet-unborn children, grandchildren, and great-grandchildren. Finance doesn’t have to be a zero-sum game if we don’t let it. If we have the right kind of financing at the right scale, we can actually do well by doing good.

This is why I’m so grateful for the opportunity to deliver this presidential address. I hope to have stimulated your interest in the emerging field of healthcare finance, and would like to pass the torch to the next generation of economists who will no doubt develop even more sophisticated financing strategies for the biomedical ecosystem of the future. That ecosystem needs more financial economists, so I hope you’ll join me in this effort.

I want to thank Katherine Virgo once more for this remarkable organization that she’s built and nurtured over decades. It’s been a pleasure and an honor to serve as president of the International Atlantic Economic Society. I congratulate Philippe Martin for being elected President-Elect, and my friend and former MIT colleague, Dimitri Vayanos, for being elected Vice President-Elect. Thank you.

## Footnotes

1 However, there is some evidence in the recent data that Eroom’s Law may be reversing (Ringel et al., 2020).

2 President Carter was treated with a combination of surgery, radiation therapy, and immunotherapy (Keytruda).

3 In fact, as of 2020Q3, the historical success rate for oncology clinical trials from phase 1 to approval is 4.0% (MIT Laboratory for Financial Engineering, 2021).

4 In 2020, $33.7 billion was invested by biotech VCs, and $22.1 billion was raised via biotech IPOs, both setting records for the global biotech industry (DeFrancesco, 2021). In 2019, global long-term bond market issuance increased by 19.7% to $21.0 trillion (SIFMA, 2020).

5 The U.S. default rates of single-A-rated bonds over the period from 1981 to 2019 were 1.53% after 9 years of issuance, 1.76% after 10 years, and 1.96% after 11 years (S&P Global, 2020, Table 25), which is consistent with our 1.82% default probability, hence our use of the single-A corporate bond yield.

6 See Hull et al. (2019) for an investigation of the feasibility of securitizing “long shots” more generally, which can be applied to other challenges that have low probabilities of success and require enormous sums of capital, but which would yield tremendous benefits if successful (e.g., climate change mitigation technologies and fusion energy).

7 See Lo and Thakor (2019) and Thakor et al. (2017) for more details on the risks and rewards of biopharma investment.

8 See MIT Laboratory for Financial Engineering (2021) for my healthcare finance publications (such as Lo et al., 2020) as well as simulation software and associated analytics for managing drug development portfolios.

9 See Wong et al. (2019) and Lo et al. (2019) for details on the methods we developed to estimate PoS.
